# Cervical human papillomavirus prevalence and genotype distribution among hybrid capture 2 positive women 15 to 64 years of age in the Gurage zone, rural Ethiopia

**DOI:** 10.1186/1750-9378-9-33

**Published:** 2014-10-08

**Authors:** Sami-Ramzi Leyh-Bannurah, Christof Prugger, Maurits NC de Koning, Hartmut Goette, Ralph J Lellé

**Affiliations:** Department of Gynecology and Obstetrics, University Hospital of Muenster, Muenster, Germany; Institute of Epidemiology and Social Medicine, University of Muenster, Muenster, Germany; INSERM, U970, Paris Cardiovascular Research Centre, University Paris Descartes, Sorbonne Paris Cité, Paris, France; DDL Diagnostic Laboratory, Rijswijk, The Netherlands; Department of Molecular Diagnostics Europe, QIAGEN GmbH, Hilden, Germany

**Keywords:** Papillomavirus infections, Cervix uteri, Ethiopia, DNA probes, HPV, Epidemiology, Risk factors, Sub-Sahara Africa

## Abstract

**Background:**

Human papillomavirus (HPV) infection is a prerequisite of cervical cancer, the leading cause of cancer mortality in Ethiopian women today. Data on Ethiopian cervical HPV prevalence and genotype distribution are rare, but essential as pre-vaccine baseline data to monitor changes after initiating HPV vaccination. The objectives of this study were to assess the cervical HPV prevalence, genotype distribution and associated correlates among female hospital outpatients in rural Ethiopia.

**Methods:**

We examined a consecutive sample of 537 women 15–64 years of age in rural Ethiopia between November and December 2006. Screening for low risk (LR) and high-risk (HR) cervical HPV infection was performed and HR positive samples were genotyped with a GP5+/6 + − and SPF10-primer based system.

**Results:**

The age-standardized prevalence of HPV, HPV HR and HPV LR infection was 17.3% (95% CI 14.1-20.5), 15.8% (95% CI 12.7-18.9) and 3.9% (95% CI 2.3-5.6), respectively. Among HC2 HPV HR positive infections (n = 86), the most common genotype was HPV 16 (24.4%), followed by 52 (11.6%), 56 (10.5%) and 31 (10.5%). Non-married relationship and widowhood, increasing number of lifetime sexual partners, human immunodeficiency virus infection and non-traditional housing type, but not age, were significantly associated with HR HPV infection.

**Conclusions:**

These results on cervical HPV prevalence and genotype distribution may serve as baseline data in evaluating the impact of future HPV vaccination programmes in rural Ethiopia.

**Electronic supplementary material:**

The online version of this article (doi:10.1186/1750-9378-9-33) contains supplementary material, which is available to authorized users.

## Background

In the year 2012, estimates of the worldwide number of cervical cancer cases and deaths amounted to 527,624 and 265,653, respectively [1]. More than 80% of global invasive cervical cancer (ICC) cases occur in developing countries and come with a higher mortality/incidence ratio [2]. National cervical cancer screening only covers less than 1% of the female population in Ethiopia, where ICC is associated with the highest cancer mortality among women [2]. For 2012, the estimated age-standardized incidence and mortality rates were 26.4 and 18.4 per 100,000 Ethiopian women, respectively, corresponding to a 4- and 9-times higher incidence and mortality rate than in Western Europe [1].

Persistent high-risk (HR) HPV infection is a prerequisite for the development of cervical cancer [3]. Because nation-wide cervical cancer screening is rarely established in countries with limited resources, HPV vaccination programmes may be an alternative and cost-effective option in these settings [4]. However, there is great heterogeneity with regard to the country-specific HPV prevalence, type-specific distribution and associated characteristics across age groups that must be considered prior to introducing HPV vaccination [5, 6]. Moreover, such baseline data are very limited in Ethiopia, but would facilitate consecutive monitoring of any changes in HPV type-specific distributions by HPV target evasion that could possibly limit the effect of HPV vaccination [7, 8].

In the present study, we report on the prevalence of cervical HPV infection, the distribution of HPV genotypes and determinants of HPV HR infection among hospital outpatients from rural Ethiopia.

## Methods

### Study population

Attat Hospital is located in the rural Gurage zone, central Ethiopia. The estimated catchment area of the hospital comprises around 1 million inhabitants [9]. Between November and December 2006, a consecutive sample of 537 women 15 to 64 years of age who visited the outpatient department were recruited with the aim of including 50 women from each 5-year age group. Main causes of outpatient visits were gynaecological diseases, perinatal care, and respiratory tract and gastrointestinal infections. Women were included if they reported to have been sexually active. Exclusion criteria were a history of hysterectomy, presence of conisation and physical or mental inability to attend the interview and pelvic examination. A total of 4 patients refused participation. At enrolment, all women provided written informed consent.

### Data collection

One senior gynaecologist performed all gynaecological examinations, and the cervix could be visualized in all women using a Cusco speculum. Cervical samples were collected using a swab (*digene* Cervical Sampler, Qiagen, Hilden, Germany) and stored at −10°C for later HPV detection and genotyping. The questionnaire was translated into Amharic and back to English to ensure equivalence. It covered sociodemographic, sexual and reproductive characteristics, and lifestyle habits. Among 235 patients (43.8%) who were not able to report their age or date of birth, age was estimated by the senior gynaecologist in consensus with the medical nurse.

### HPV screening

The *digene* Hybrid Capture 2 HPV DNA Test ([HC2] Qiagen, Hilden, Germany) was performed according to manufacturer’s specifications at the Cytopathology Laboratory of the Department of Gynaecology and Obstetrics, University of Muenster, Germany, as described previously [10]. Using the HR HPV probe, 13 HR HPV types (16/ 18/ 31/ 33/ 35/ 39/ 45/ 51/ 52/ 56/ 58/ 59/ 68) can be collectively detected without identifying the HPV genotype. Similarly, five low-risk (LR) HPV types (6/ 11/ 42/ 43/ 44) can be detected with the LR HPV probe.

**Figure 1:**
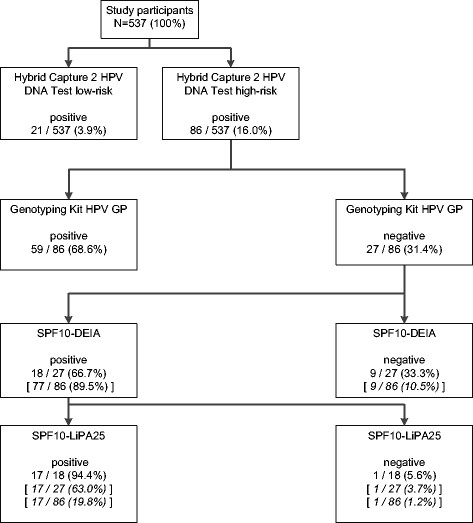
**Flow diagram of sample processing.** HPV screening by Hybrid Capture 2 HPV DNA Test, followed by β-Globin-PCR testing (not shown in figure) and high risk HPV genotyping of all HC2 HR positive samples by the Genotyping Kit HPV GP and subsequent testing by the SPF10-LiPA25 system, version 1, of all HC2 HR HPV positive samples, in which no HPV type was identified by the Genotyping Kit HPV GP. Square brackets show the number (%) of results in relation to the preceding assay performed.

All HC2 HR HPV positive samples were further analysed at DDL Diagnostic Laboratory (Rijswijk, Netherlands). An overview of the HPV detection and consecutive HPV genotyping algorithm is shown in Figure 1.

### HPV genotyping

The quality of the isolated DNA was checked by amplifying a 268 base pair fragment from the ß-globin gene with primers PC04 and GH20 [11]. First, the Genotyping Kit HPV GP (Diassay BV, Rijswijk, The Netherlands) was used according to the instructions of the manufacturer [12]. This kit identifies 14 HR genotypes (16/ 18/ 31/ 33/ 35/ 39/ 45/ 51/ 52/ 56/ 58/ 59/ 66/ 68 with subtype 68a) and 4 possible HR HPV types (26/ 53/ 73/ 82 with subtypes IS39 and MM4). Second, in HC2 HR HPV positive samples in which no HPV type was identified by the Genotyping Kit HPV GP (n = 27; Figure 1), the highly sensitive SPF10-LiPA25, version 1 system (Labo Bio-medical Products, Rijswijk, The Netherlands) was used for further evaluation of HPV genotypes as described previously [13]. The SPF10-DNA Enzyme Immuno Assay (DEIA) allows a pooled broad spectrum HPV detection of more than 54 HPV types and the SPF10-line probe assay (LiPA) enables subsequent genotyping of 25 different LR and HR HPV genotypes (6/ 11/ 16/ 18/ 31/ 33/ 34/ 35/ 39/ 40/ 42/ 43/ 44/ 45/ 51/ 52/ 53/ 54/ 56/ 58/ 59/ 66/ “68/73”/ 70/ 74). Genotypes 68 and 73 cannot be discriminated by the LiPA [13].

### Statistical analysis

Descriptive statistics are presented as numbers (%) and median (interquartile range, IQR). Prevalence estimates were age standardised using the world standard population as reported by Doll et al. [14]. Associations of patient characteristics with HPV HR infection were assessed using logistic regression analysis to estimate odds ratios (ORs) with 95% confidence intervals (CI). A multivariable model was fitted using backward selection among variables identified in univariate analyses using an entry threshold of p < 0.10. All tests were two-sided and an alpha level of 0.05 was chosen to indicate statistical significance. Analyses were performed using SPSS 18.0.3 (IBM, New York, USA).

## Results

### Patient characteristics

Median age at onset of menstrual bleeding was 13 years (IQR 13–14), age at sexual debut and first pregnancy were both at 18 years (IQRs 15–20 and 15–21, respectively). The median number of life births reported was 4 (IQR 2–7).

Overall, 391 (72.8%) women were in a monogynous marriage and 102 (19.0%) were widowed. Median length of partnership was 9 years (IQR 3–19). The median number of lifetime sexual partners was 1 (IQR 1–1), with 123 (22.9%) women having more than one lifetime sexual partner. 404 (75.2%) patients denied to have ever used any contraceptive method.

### Frequency of HPV infections

Figure 1 shows the flow chart of sample processing. The frequency of HPV HR and LR infection was 16.0% and 3.9%, respectively. A HPV HR and LR co-infection was present in 13 (2.4%) women. Thus, a total of 94 (17.5%) women were classified as HPV positive by HC2. The age-standardized prevalence of HPV, HR HPV and LR HPV infection amounted to 17.3% (95% CI 14.1-20.5), 15.8% (95% CI 12.7-18.9) and 3.9% (95% CI 2.3-5.6), respectively.

**Figure 2:**
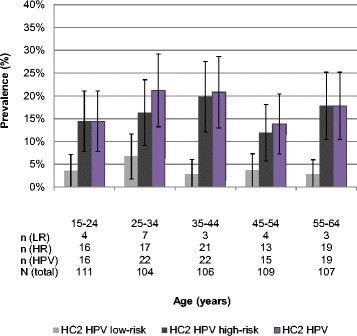
**Age-specific prevalence (%) of cervical human papillomavirus infection.** Low risk, high risk and overall HPV prevalence and corresponding 95% confidence intervals according to ten-year age groups among 537 women, Gurage zone, rural Ethiopia. Results are based on DNA detection by Hybrid Capture 2 DNA test.

Figure 2 shows the frequency of HPV, HR HPV and LR HPV infections by 10-year age groups. Neither the frequency of HPV, nor the one of HR HPV and LR HPV infections showed a significant age pattern (p for trend = 0.473, 0.566 and 0.571, respectively).

### HPV genotypes

All 86 HC2 HR HPV positive samples were used for subsequent HPV genotyping. As shown in Figure 1, primary testing by the Genotyping Kit HPV GP identified the HPV type in 59 samples (68.6%). Further testing with the highly sensitive SPF10-LiPA25, version 1, additionally identified 15 HR HPV (partially with HPV co-infections) and two LR mono-infections. Despite being SPF10 DEIA positive, one sample remained LiPA negative, indicating the presence of another type than the 25 HPV types included on the LiPA. An exact genotype could not be identified.

Table 1 presents the cervical HPV genotype distribution identified by Genotyping Kit HPV GP and SPF10-LiPA25 system, version 1, among HC2 HR HPV positive infections (n = 86). Table 2 allows discriminating between the genotyping results of both PCR assays. Among HC2 HR HPV positive infections (n = 86), the most common genotype was HPV 16 (24.4%), followed by 52 (11.6%), 56 (10.5%), 31 (10.5%), 51 (7.0%), 35 and 39 (both 5.8%), 45 and 68 (both 4.7%), and 18 (3.5%). Overall, 17 different HR and 3 different LR HPV genotypes were identified, amounting to a total of 92 HR or LR infections. In this group, 49 (53.3%), 19 (20.7%) and 12 (13.0%) HPV infections were attributed to species α9 (HPV 16, 31, 33, 35, 52, 58, 67), α7 (HPV 18, 39, 45, 59, 68, 70) and α6 (HPV 53, 56, 66), respectively, based on their phylogenetic classification [15]. Furthermore, in this group, 24 (26.1%) and 52 (56.5%) HPV infections would be targeted by a current bivalent (types 16 and 18) and an impending next generation nonavalent (types 6, 11, 16, 18, 31, 33, 45, 52 and 58) HPV vaccine, respectively [16].

**Table 1:** **Cervical HPV genotype distribution and phylogenetic classification among HC2 high risk positive women, Gurage zone, rural Ethiopia**

HPV genotypes*	Number of HPV infections			n = 86	Phylogenetic classification
	Single	Multiple**	Total	Percent	Species
11^a^	1	0	1	1.2	α10
16	16	5	21	24.4	α9
18	1	2	3	3.5	α7
31	6	3	9	10.5	α9
33	1	1	2	2.3	α9
35	4	1	5	5.8	α9
39	4	1	5	5.8	α7
43^a^	0	1	1	1.2	α8
45	3	1	4	4.7	α7
51	5	1	6	7.0	α5
52	7	3	10	11.6	α9
53	1	0	1	1.2	α6
56	5	4	9	10.5	α6
58	2	0	2	2.3	α9
59	1	0	1	1.2	α7
66	2	0	2	2.3	α6
68	2	2	4	4.7	α7
70^a^	1	1	2	2.3	α7
73	0	1	1	1.2	α11
82	1	0	1	1.2	α5
68/73^b^	2	0	2	2.3	α7 or α11

*Genotypes identified in HC2 high risk positive infections by Genotyping Kit HPV GP and SPF10-LiPA25, version 1.

**Only HPV high risk co-infections were considered.

^a^HPV low-risk genotype.

^b^Discrimination between HPV genotypes 68 and 73 is not possible with SPF10-LiPA25 system, version 1.

**Table 2:** **Cervical HPV infection stratified by results of Genotyping Kit HPV GP and SPF10-LiPA25, version 1 among HC2 high risk positive women, Gurage zone, rural Ethiopia**

HPV infection	Total	HPV genotype by genotyping Kit HPV GP	HPV genotype by SPF10-LiPA25, ver. 1*
	n	n	n
11^a^	1		1
16	16	16	
18	1	1	
31	6	6	
33	1	1	
35	4	4	
39	4	2	2
45	2	2	
51	5	1	4
52	7	4	3
53	1		1
56	5	5	
58	2	1	1
59	1	1	
66	2	2	
68	2	2	
70^a^	1		1
82	1	1	
68/73^b^	2		2
16,56	2	2	
18,52	1	1	
18,56	1	1	
31,56	1	1	
31,68	1	1	
39,73	1	1	
45,70^a^	1		1
16,31,35	1	1	
16,33,68	1	1	
16,45,52	1	1	
43^a^,51,52	1		1

*Only samples negative by Genotyping Kit HPV GP were tested by SPF10-LiPA25, version 1.

^a^HPV low-risk genotype.

^b^Discrimination between HPV genotypes 68 and 73 is not possible with SPF10-LiPA25, version 1.

### Univariate and multivariable associations with HPV HR infection

In univariate analyses for the association with HPV HR infection, the following patient characteristics did not reach the threshold of p < 0.10: religion, educational level of the women and their partner, patient’s income, age at menarche, parity, current pregnancy, female circumcision, alcohol consumption and khat chewing (Additional file 1). Table 3 presents univariate and multivariable associations with HPV HR infection of the remaining patient characteristics under investigation: non-married relationship and widowhood, increasing number of lifetime sexual partners, human immunodeficiency virus infection (HIV) and non-traditional housing type were independently associated with HR HPV infection.

**Table 3:** **Odds ratios for HR HPV positivity and corresponding 95% CIs by selected characteristics, Gurage zone, rural Ethiopia**

	HC2 HR positivity		Univariable			Multivariable		
	n/total	%	OR	95% CI	p-value	AOR	95% CI	p-value
**Age interval**								
15-24	16/111	14	1.00		*0.572*			
25-34	17/104	16	1.16	(0.55 - 2.44)	*0.695*			
35-44	21/106	20	1.47	(0.72 - 2.99)	*0.292*			
45-54	13/109	12	0.80	(0.37 - 1.76)	*0.586*			
55-64	19/107	18	1.28	(0.62 - 2.65)	*0.502*			
**Marital status**								
Married, monogynous	51/391	13	1.00		*0.021*	1.00		*0.047*
Married, polygynous	4/18	22	1.91	(0.60 - 6.01)	*0.272*	1.26	(0.35 - 4.57)	*0.728*
With Partner (not married)	3/6	50	6.67	(1.31 - 33.93)	*0.022*	7.22	(1.37 - 38.10)	*0.020*
Divorced or separated	4/20	20	1.67	(0.54 - 5.18)	*0.378*	0.66	(0.16 - 2.69)	*0.561*
Widowed	24/102	24	2.05	(1.19 - 3.53)	*0.010*	1.85	(1.01 - 3.41)	*0.047*
**Lifetime number of sexual partners**								
1	55/414	13	1.00		*0.004*	1.00		*0.011*
2	25/106	24	2.01	(1.18 - 3.43)	*0.010*	1.83	(1.03 - 3.23)	*0.039*
≥ 3	6/17	35	3.56	(1.27 - 10.02)	*0.016*	3.94	(1.33 - 11.65)	*0.013*
**History of STD**								
Never	71/502	14	1.00		< *0.001*	1.00		*0.002*
Other STD than HIV*	4/14	29	2.43	(0.74 - 7.95)	*0.143*	1.95	(0.57 - 6.69)	*0.291*
HIV	9/12	75	18.21	(4.81 - 68.90)	< *0.001*	13.59	(3.25 - 56.89)	*< 0.001*
Unknown/unsure	2/9	22	1.73	(0.35 - 8.52)	*0.498*	2.47	(0.49 - 12.45)	*0.274*
**Main house**								
Traditional thatched house (gojo)	60/429	14	1.00			1.00		
Other	26/108	24	1.95	(1.16 - 3.28)	*0.012*	1.98	(1.10 - 3.57)	*0.022*

Results are based on high risk HPV DNA detection by Hybrid Capture 2 DNA test and presented among 537 women.

*Other STDs reported were gonorrhea, syphilis and chlamydia infection; 95% CI = 95% confidence interval; AOR = adjusted odds ratio; HC2 = hybrid capture 2 HPV test; HIV = human immunodeficiency virus; OR = odds ratio; STD = sexually transmitted disease.

## Discussion

The present study reports on the prevalence of cervical HPV infection, the distribution of HPV genotypes and determinants of HPV HR infection in rural Ethiopia. Our study reveals several important findings.

### HPV prevalence

We observed an age-standardised prevalence of HPV infection of 17.3% and an age-standardised prevalence of HPV HR infection of 15.8%. A review by Smith et al. reported great variations in the HPV prevalence across African countries ranging from 12% to 46% [17]. Specifically, studies from the Sub-Saharan African countries Nigeria [18], Uganda [19] and Mozambique [20] reported HPV HR frequencies of 14.7%, 19.2% and 40.0%, respectively. In comparison, HR HPV prevalence estimates for developed countries in Western Europe range from 9.4% to 12.1% [21]. Thus, our study results indicate that the HPV HR prevalence in rural Ethiopia is consistent with reports from Sub-Saharan Africa, but almost twice as high as in most Western European countries.

No significant age pattern was observed, despite including the rarely reported age interval of 15 years up to 64 years in our study. However, instead, there was a rather high HPV HR prevalence of at least 10% in each 10-year age group. Worldwide and African studies reported heterogeneous age patterns: HPV HR prevalence decreasing with age [5, 19, 22], showing an U-shaped curve [22, 23] or being consistently high across all age groups [5, 20, 24]. The latter is the case in the present study, possibly indicating a lifespan HPV persistence and/or a reacquisition with increasing age due to changing sexual behaviour and age-related changes of mucosal biology and immune competence [5, 23]. This is also consistent with our result on widowhood, which increased with age, as a correlate for HR HPV infection.

### HPV genotypes

The 5 most common HPV genotypes in our study sample were HPV 16 (24.4%), followed by 52 (11.6%), 56 (10.5%) and 31 (10.5%) and 51 (7.0%). Among women with normal cytology, HPV types 16, 18, 31, 58, 52 and 52, 16, 18, 53, 66 are estimated to be the most common worldwide and in Eastern Africa, respectively [22]. Our study reveals HPV genotypes 16 and 52 as predominant genotypes in Ethiopia. HPV 16 has the highest carcinogenic potential and is targeted by current HPV vaccines [15]. Nonetheless, as expected, also oncogenic HR HPV genotypes were identified, which are not targeted by current and advertised polyvalent vaccines [16]. However, HPV vaccination remains a promising approach in Ethiopia for multiple reasons. First, vaccines may provide cross-protection against non-vaccine type HPVs [25, 26]. Second, many oncogenic HPV infections that evade the vaccine target range are transient and do not reach relevant stages of cervical carcinogenesis [27]. Third, specifically in settings like Ethiopia with very limited resources and poor cervical cancer screening coverage, HPV vaccination may represent a definitive prevention strategy. Our data on Ethiopian HPV prevalence and genotype distribution would represent a first step to build baseline data enabling to monitor dynamics in HPV genotype distribution as an effect of HPV vaccination that can be registered earlier than changes in (pre)cancerous cervical lesions, which naturally can take decades. However, Ethiopian data collection on baseline HPV type prevalence in ICC patients also remains a priority so that long term HPV vaccine efficacy can be finally evaluated.

In our study, HPV types 18, 53 and 66 were not corroborated as other common types in Eastern Africa, but were rather rare [22]. First, this might be attributed to the low share of African studies in worldwide reviews, i.e. only 3.9% in a meta-analysis by de Sanjose et al. [22] and thus limited data from African regions. Second, HPV results from various populations often differ by age ranges, settings and HPV assays used [28], which makes comparisons difficult. However, our study includes a wide age range (15–64 years), a balanced age distribution of at least 50 participants per 5-year age group and established HC2 HPV screening with a sophisticated two-step genotyping algorithm.

### Correlates of HC2 HR HPV infection

Our analyses revealed that non-married relationship and widowhood, increasing number of lifetime sexual partners, human immunodeficiency virus infection (HIV) and non-traditional housing type were independently associated with HR HPV infection. These results are in line with previous reports showing that marital status, number of lifetime sexual partners and history of STDs, particularly HIV, are established risk factors for HR HPV infection [3, 19, 28, 29].

A housing type other than gojo, presumably depicting a higher socioeconomic status or an urban living area, was positively associated with HPV HR infection. Conversely to this finding, many studies have described an increased risk of HPV infection with lower socioeconomic status [30]. Possibly, living in rural areas can be beneficial with regard to female health status in our study setting. Supporting this theory is a 11-fold difference in HIV prevalence between rural and urban areas in Ethiopia (0.7% versus 7.7%, respectively) [31]. Neither parity nor age at first pregnancy nor use of oral contraceptives was significantly associated with HPV HR infection. These results are consistent with a meta-analysis by the International Agency on Research of Cancer [32].

### Sample testing

In our study, primary screening was performed with HC2 because of high clinical sensitivity and specificity [33]. The occurrence of discordant results in our assay algorithm (i.e., HC2 positive with no HR HPV identified) may be attributable to false positive results with HC2 as exemplified by the 2 LR HPV mono-infections [34–36]. This would explain negative results in the subsequent Genotyping Kit HPV GP. Another possible explanation for a discordant result could be a sequence variation in the L1 target area, disrupting the GP5+/6 + − PCR target [37]. Because of the very high analytical sensitivity, we used the SPF10-LiPA25, version 1, for discrepancy analysis between the Genotyping Kit HPV GP and HC2 [13, 34], resulting in additional 17/27 (63.0%) genotyped samples. This may be related to the superior analytical sensitivity of the SPF10-LiPA25, version 1, compared to GP5+/6 + −primer based assays [13].

### Limitations and strengths

Our study has certain limitations. First, patients were recruited in the single setting of Attat hospital and morbidity may be higher than in the general population. However, representative Ethiopian data are currently lacking, making our report the most comprehensive on HPV prevalence in Ethiopia yet. Second, no cytological evaluation was performed that would allow further sample stratification. However, this study focussed on HPV DNA testing in order to assess the burden of HPV infection and build baseline data prior to the implementation of HPV vaccination programmes to allow monitoring eventual changes in HPV prevalence and genotype distribution.

Third, the restriction of HPV genotyping to HC2 HR positive samples might have led to a bias in HPV type distribution and presentation.

### Perspectives

Health economic models for evaluating the potential benefit of implementing HPV vaccination programs indicate that such interventions may be very cost-effective in Eastern Africa [4]. Challenges in the implementation must be considered, such as vaccine delivery (e.g. through health centers and schools), vaccine effect monitoring (e.g. sampling method, laboratory infrastructure, means of registration) and a vast cultural and regional diversity within Ethiopia. Corresponding analyses for Ethiopia are currently lacking and should thus be conducted before HPV vaccine delivery can finally be advocated. If Ethiopian large-scale introduction of HPV vaccination is envisaged, our data on the HPV genotype distribution would enable monitoring associated benefits and harms in rural settings.

## Conclusion

Our results show a high frequency of at least 10% HPV HR infection across all age groups, which is an important risk factor for cervical cancer development. The predominant HPV genotype that is already targeted in current HPV vaccination was 16 (24.4%). For future use of HPV vaccines, our study provides the first share of important baseline data that would enable monitoring of interim HPV vaccination effects, such as changes in HPV prevalence and genotype distribution.

### Ethical approval

The ethics committee of the Medical Association Westfalen-Lippe and the Medical Faculty of the University of Muenster, Germany, approved the study.

## Electronic supplementary material

Additional file 1: **Odds ratios for HR HPV positivity and corresponding 95% CIs by remaining characteristics, Gurage zone, Ethiopia.** The characteristics shown did not reach the threshold level for multivariable analysis. (XLSX 22 KB)

## Abbreviations

CI: Confidence interval
DEIA: DNA enzyme immuno assay
HC2: Hybrid capture 2 HPV DNA test
HR: High risk
HPV: Human papillomavirus
ICC: Invasive cervical cancer
LR: Low risk
LiPA: Line probe assay
STD: Sexually transmitted disease.
